# Atlanta Society of Medicine

**Published:** 1894-02

**Authors:** R. R. Kime

**Affiliations:** Secretary; Atlanta, Ga.


### *SOCIETY REPORTS.*

---

### ATLANTA SOCIETY OF MEDICINE.

ATLANTA, GA., January 16th, 1894.

*J. W. Duncan, M. D., President, in the Chair.*

Subject for discussion—

### ERGOT IN OBSTETRICS.

(SYNOPSIS OF DISCUSSION OPENED BY DRS. HARDON AND TODD.)

DR. HARDON: This subject is of personal interest. I do not propose to dogmatize or ride a hobby but present views which have developed in a practice of twenty years. At the time I commenced practice a bottle of ergot was in every obstetric satchel and used as a routine practice. Later from experience and practice I have abandoned its use.

Its physiological effect is most marked on the pregnant uterus, having but slight effect on the non-parturient. It acts on muscular fibers of uterus in cervix as well as body of uterus producing tonic contractions, not a normal contraction but one that lasts until effect of drug passes off. Those that advocate its use give it only when os is dilated or dilatable with a presentation that will not interfere with delivery and no mechanical obstruction present. This limits its use to cases of uterine inertia and leaves out the first stage of labor. Its effect in second stage of labor, head in womb or in vagina, is a tonic contraction imprisoning fetus in uterus, or cervix around neck and impedes delivery. By such contraction the blood is squeezed out of placenta and child fails to get necessary amount of oxygen and dies often. Some claim ergot is liable to produce inflammation of the womb by its prolonged effect, contractions, etc.

In third stage of labor ergot contracts cervix and imprisons placenta, prevents its expulsion or delivery. I have seen cases where cervix was so firmly contracted from use of ergot two fingers could not be passed into uterus and would have to wait for effect of drug to pass off. It produces what is often mistaken for hour-glass contractions of uterus, which is simply a contraction of internal os. Ergot destroys physiological law of uterus, viz.: when body contracts cervix relaxes ; when cervix contracts body relaxes. Ergot does not always produce expulsion of uterine contents but imprisons it.

Dangers from its use:

1. Death of child.
2. Imprisonment of child.
3. Inflammatory process in uterus.
4. Hour-glass contractions of uterus.
5. Retention of placenta, blood-clots, etc.

With dilated cervix, normal presentation and inertia of uterus, forceps is better than ergot and safer. The child's chances for life are better with use of forceps than with ergot. In post-partum hemorrhage do not wait to use ergot. Clear out uterus, with one hand in uterus the other on abdomen, knead uterus and make it contract.

DR. TODD: We do not disagree very much. I will present my experience, honestly culled and honestly given:

Ergot should not be given in shoulder presentations, rigid perineum, etc. With a normal presentation, dilated or dilatable os, ergot can be used to advantage in uterine inertia. The difficulty in arriving at the physiological effect of the drug is that most authorities make their observations from large doses, ʒ j. For the last eighteen years I have not used any but Squibbs's aqueous extract, which is reliable and satisfactory. Drachm doses are too large and will produce tetanic contractions of the uterus. I give it in small doses, five to ten minims every ten to fifteen minutes, which do not produce tetanic contractions but the normal contractions with increased force.

Glad war was waged against large doses of ergot. The majority of the profession do not know how to use forceps. Gross was right when he said never take forceps with you to an obstetric case, as you will have plenty of time to send for them when needed. Give medicinal, not poisonous, doses of ergot. Roosevelt Hospital gives ergot as a routine after labor. Large doses, long contractions result and may favor retention of placenta but small doses will not. In post-partum hemorrhage clean out uterine cavity and keep hand in uterus until it contracts, then ergot, in large doses, is the thing to give; tetanic contraction is what is needed. In small doses will expel blood-clots, not in large doses. Forceps, with those who know how to use them, for inertia, then follow with ergot to prevent hemorrhage. There are but very few physicians I would trust to use forceps. Would prefer, where conditions are favorable, the use of ergot in small doses.

GENERAL DISCUSSION.—Dr. Gaston thinks Dr. Hardon's views mostly theoretical. Have used ergot for years, formerly the crude drug with cinnamon tea. Had no untoward effect, rhythmical contractions, did not give it until os was dilated. Never saw a case of hour-glass contraction where he had given ergot, but had seen some twenty-five or thirty cases where ergot was not given. Hour-glass contraction occurs in body of uterus and is overcome by pressure. In his earlier years of practice, when ergot was used, saw but few cases of lacerated perineums. Of late years used forceps in place of ergot. Differs with Dr. Hardon and advocates its use in post-partum hemorrhage.

DR. OLMSTED gives ergot but seldom, cannot see why it should not be used in normal presentation, os dilated and uterine inertia present. Uses it in post-partum hemorrhage, and especially in those with predisposition to hemorrhage uses Squibbs's preparation. Never had retained placenta or hour-glass contraction from ergot. In post-partum hemorrhage clean uterine cavity with hand, then give ergot. After use of forceps for uterine inertia thinks ergot useful. Has used quinine, ten to fifteen grains, in four cases, with benefit.

DR. FLOYD W. MCRAE had used ergot but little, never before labor was completed; had given it a few times after labor. Saw one case where he thought ergot caused death by contraction of arterioles. It contracts arterioles, nervous system, brain, etc., as well as womb, and is certainly dangerous where hemorrhage is severe. Never had a case of hour-glass contraction. In post-partum hemorrhage thoroughly empty uterus and it will contract. Forceps properly used will not rupture perineum and is preferable in uterine inertia to use of ergot. Agrees with views expressed by Dr. Hardon to-night.

DR. CROWE: Experience varied and in a measure agreed with both views expressed to-night. First five years of practice used ergot freely, later used it less. In fleshy women, flabby muscular structures, with tendency to hemorrhage, advises ergot. Never gives it before birth of child, is not safe nor rational. Its use before birth of child will rupture more perinea than forceps. Has seen numbers of cases of ruptures result from the use of ergot in this city. Has used quinine with good results.

DR. F. B. McRAE uses ergot for inertia, prefers small doses. Had not seen ruptured perineum but retained placenta from its use. Do not give until placenta is expelled.

DR. KIME: Very much interested in the discussion. Was imbued with the idea that the obstetric bag should contain ergot, and in the first seven or eight years of his practice used it quite frequently, but in the last eight years has gradually abandoned its use, believing that we have other remedies more efficient and less dangerous.

It is no theory but a fact which is demonstrated by experience that ergot not only contracts muscular fiber of the body of uterus but also of the cervix, especially the circular fiber at internal os and those of external os when not lacerated, thus preventing drainage or emptying of the contents of the uterus. Had administered it in small doses after labor with a view of lessening size of uterus and hastening involution but had seen putrid infection as a result of its use. It contracts internal os, shuts up and retains blood-clots and secretions which decompose and are absorbed. Had seen such cases and dilated cervix with steel dilators, allowing from one to several ounces of purulent putrid material to escape, using irrigation, disinfection and drainage for relief of patient. In uterine inertia quinine, whisky, strychnia and forceps are far preferable to ergot. Has used quinine and whisky to increase labor pains for years, and in uterine inertia prefers them or the use of forceps in preference to ergot. That ergot given before the birth of child is liable to produce its death, lacerate cervix or perineum; given afterwards interferes with delivery of placenta, prevents drainage and favors infection of patient by retention, decomposition and absorption of uterine contents.

DR. HURT uses it when indicated. Thinks the difficulty is due to injudicious use and dose. Uses it for inertia when cervix dilated, and normal presentation given it with whisky. Had seen a case of convulsions a result of its use; also a lacerated perineum. Had seen hour-glass contractions in a dozen or more cases where ergot was not used. Does not believe ergot will cause retention of placenta, blood-clots, etc. Uses forceps often for inertia. For post-partum hemorrhage clean out uterus with hand, then give ergot.

PRESIDENT does not use it before child is born, but frequently gives it after labor. Prefers forceps for inertia. For post-partum hemorrhage would not wait for ergot; runs hand into uterus, cleans out, using hot water, ice, and later may give ergot.

DR. HARDON: My views are the result of years of practical experience. It is no theory, but a fact, capable of demonstration, that ergot will imprison fetus, placenta, blood-clots, etc. It seems all advocates of the use of ergot are agreed to-night that the field for its use is very small, indicated only in uterine inertia, with a normal presentation and dilated os, where no mechanical obstruction to delivery exists, used in post-partum hemorrhage after it is checked by other measures; so in the majority of instances, where assistance is needed, we must depend on something besides ergot.

I see before me to-night physicians who have had retained placenta from the use of ergot. I see one who lost a case from septicemia, the result of use of ergot. Have seen cases die in this city from use of ergot.

DR. TODD: This discussion will result in good. I hope I did, not create the impression that I used ergot in every case, for I have used it but few times before birth of child. Give quinine and rarely resort to ergot. The only case of hour-glass contraction I ever had was result of ergot. I would risk but three present to-night to use forceps for me. If the master was always present, then they might take the place of ergot, but there are very few physicians that know how to use forceps.

In hemorrhage do not discard ergot because it contracts arterioles, but guard it with nitroglycerine. Do not wait for ergot in post-partum hemorrhage, but after uterus is emptied it is the very thing needed. Ergot is often a good remedy to prevent convulsions.

R. R. KIME, M. D., Secretary.

